# Consumption Context Effects on Fine Wine Consumer Segments’ Liking and Emotions

**DOI:** 10.3390/foods9121798

**Published:** 2020-12-03

**Authors:** Lukas Danner, Trent E. Johnson, Renata Ristic, Herbert L. Meiselman, Susan E.P. Bastian

**Affiliations:** 1 School of Agriculture, Food and Wine, Waite Research Institute, The University of Adelaide (UA), PMB 1, Glen Osmond 5064, Australia; lukas.danner@adelaide.edu.au (L.D.); trent.johnson@adelaide.edu.au (T.E.J.); renata.ristic@adelaide.edu.au (R.R.); 2 Herb Meiselman Training and Consulting Services, Rockport, MA 01966, USA; herb@herbmeiselman.com

**Keywords:** Australian wine evoked emotions lexicon (AWEEL), consumer behaviour, consumer segmentation, emotions, hedonic, fine wine instrument

## Abstract

Wine consumer lifestyle segmentation has been widely studied; however, most studies have solely utilised online surveys. This work investigated the impact of context on wine consumer segments’ liking and emotions while consuming wines in different environments. Two studies were conducted with regular wine consumers segmented based on their fine wine behaviour using the Fine Wine Instrument. Study 1 (*n* = 122) investigated the effects of wine variety and product information, and Study 2 (*n* = 346) the effects of wine quality and consumption context, on hedonic and emotional responses of the segments. Within both studies, three segments were identified and named: Wine Enthusiasts, Aspirants and No Frills. The Wine Enthusiast segment generally liked the wines more and perceived more intense positive emotions when consuming wine compared to the No Frills segment, with the Aspirant’s likes and emotion intensities ranging in between. Wine Enthusiasts were more discriminative of their preferred wines and reported stronger positive emotions when tasting higher quality (Study 1) and more complex (Study 2) wines. The consistent results across the two studies showed for the first time that consumer segments, based on lifestyle segmentation, differ in their hedonic and emotional responses towards wine when actually tasting wines, demonstrating that the Fine Wine Instrument has practical implications and can identify wine consumers displaying different wine consumption behaviours.

## 1. Introduction

Market segmentation has been used as a strategy by companies since the 1950s to identify homogeneous groups of consumers within a heterogeneous market and thereby provide a competitive advantage, such as tailored products and marketing communications that meet the needs of the identified consumer segments [1,2,3,4]. The Australian wine market has a long history of academic study into market segmentation (see, for example [5,6,7,8,9,10,11,12,13,14,15,16,17]).

However, it has been a continuing challenge to identify the most suitable segmentation basis for this heterogeneous market [18] as traditional approaches, such as those based on demographics, have been questioned due to a lack of richness [19,20,21]. In recent years, “domain-specific” lifestyle segmentation (i.e., consumer segmentation based on psychographic data specific to a specific consumption situation or a set of consumption behaviours) has been identified as a promising segmentation method for food in general [21] and specifically for wine (for example [14,15,22,23]). For more detailed analyses of the history of wine market segmentation and the various segmentation bases available, see Johnson and Bastian [8] and Johnson, Danner and Bastian [24,25].

One such lifestyle segmentation study was that of Johnson and Bastian [8], who argued that a multi-dimensional psychographic segmentation tool based on Attitudes, Interest and Opinions (AIO) would provide a more detailed and sophisticated description of any identified segments, than more traditional segmentation bases. They developed the Fine Wine Instrument (FWI) that consists of 18 scale items covering three dimensions, labelled connoisseur, knowledge and provenance. These three dimensions then formed the segmentation base for a cluster analysis that identified and characterised three fine wine-related segments in the Australian wine market.

Although there are numerous studies applying “domain-specific” lifestyle segmentation in different markets [24,25,26,27], all published studies solely relied on respondents’ self-reported answers collected through online surveys or face to face questionnaires. None of these studies investigated consumers within real life tasting situations nor how their responses differed by wine-related behaviour-based segments when actually tasting wines. The aim of the current research was to investigate consumer behaviour when tasting wines in different consumption contexts, consistent with the opinions of Jaeger et al. [28] and Meiselman [29], who advocated for research in more complex consumption situations as means to improve ecological validity over an unnatural sensory laboratory setting.

While product aroma and flavour characteristics are essential sensory elements underpinning consumers’ preferences and purchase decisions [30,31], consumer perceptions, liking and decision making are also influenced by the context of consumption [32,33,34,35]. Context considers social and environmental factors plus the product itself, including not only intrinsic attributes but also extrinsic characteristics such as the product related information provided at consumption [34]. It has been shown that both experts and novice consumers rely on extrinsic cues when evaluating wine [36] and that the consumption situation (different locations, wine with or without food) can influence consumers’ liking [37,38] and more general perception [35] of wines.

The product information available to the consumer when tasting or consuming a wine, e.g., that presented on wine labels, can also be regarded as a contextual factor and has the potential to influence their expectations [39] and hedonic responses [40,41]. D’Alessandro and Pecotich [36] showed that when choosing, and judging wine quality and price, both wine experts and novices were influenced by extrinsic cues. However, little research has been published investigating context effects on different wine consumer segments as opposed to a heterogeneous consumer cohort, resulting in the first research question: Do the hedonic responses of fine wine segments (FWS) generated by the FWI differ when tasting wines of diverse varieties or quality levels in different consumption contexts?

In the past decade, research has shown that hedonic responses (i.e., liking) are only one component of the wine consumption experience and that consumption evoked emotions can provide additional insights into consumers’ perceptions and product related behaviour [42,43,44,45]. More recently Calvo-Porral et al. [46] used consumers’ emotional associations with wine consumption as a segmentation basis and identified four distinct clusters: the unattached, the negatives, the circumspect and wine lovers. This raises the second research question: Do reported wine-evoked emotions differ between FWS?

## 2. Materials and Methods

To address the two research questions and to investigate if the effects are consistent across multiple studies and wine varieties, participants of one white wine and one red wine study were segmented using the fine wine instrument and their hedonic and emotional responses during actual wine consumption analysed. Study 1 [41] investigated the effects of intrinsic and extrinsic product context factors on liking and emotional responses for the three FWS by evaluating three different white wine varieties presented together with three levels of product information. Study 2 [47] investigated how intrinsic product and environmental context factor impact on the FWS’s hedonic and emotional responses when tasting Shiraz wines of differing quality levels in three separate consumption contexts (sensory laboratory, restaurant and at home). Furthermore, moods in anticipation of the wine tasting were assessed for the FWS.

Full materials and methods details can be found in [41,47] while all relevant details regarding the present research and data analyses are stated here.

All studies were performed in accordance with the ethical guidelines for scientific research at the University of Adelaide and approved by the human ethics committee (approval numbers: H-2013-048). Participants gave written informed consent prior to the tasting.

### 2.1. FWI and Emotion Scales

The 18 item Fine Wine Instrument Scale, comprised of 3 dimensions, connoisseur behaviour, wine knowledge and wine provenance, was used for consumer segmentation (Appendix A Table A1) [8]. This formed the basis for the Agglomerative Hierarchical Clustering (AHC) employed in the two studies described below. Emotions were measured on a 9-point Likert scale using AWEEL [47]. To assess consumers’ wine liking a 9-point hedonic scale was used ranging from 1 = dislike extremely to 9 = like extremely.

### 2.2. Study 1 (White Wines)

#### 2.2.1. Wine Samples

The aim was to select wines that were distinctly different from each other and cover different styles. Three commercially available Australian white wines, one Chardonnay (oaked), a Riesling and a Sauvignon Blanc, being the most commonly consumed white wine varieties in Australia, were selected by four wine experts (as defined by Parr et al. [48]). Additionally, an unoaked Chardonnay was used as a warm-up sample to familiarise participants with the tasting procedure, as a control for first position order effects and to allow comparison between the two tasting sessions. Based on the results of a descriptive analysis [49] of the wines, two wine descriptions were developed for each wine; a basic description, exclusively based on the objective sensory profile and a more elaborate description using more emotive sensory terms and including information about wine quality and background information of the winery (the full descriptions can be found in Danner et al. [41]).

#### 2.2.2. Tasting Procedure

In total, 126 regular white wine consumers (who consumed white wine at least once a month) evaluated the three wines under three context/information conditions, blind (no information provided), basic, and elaborate wine descriptions (for full descriptions see Danner et al. [41]). Consumers completed an online recruitment questionnaire including basic demographic data, wine consumption behaviour and the FWI scale. To conceal the purpose of the experiments, the tasting was split across two sessions, at least one week apart, and consumers were informed that they were participating in two unrelated studies. In session 1, participants tasted the wines under blind conditions and in session 2, tasted wine together with the more detailed wine information. Then, 30 mL of samples were presented in coded ISO tasting glasses at 12–13 °C randomised within each session across participants. The sensory sessions were held in individual sensory booths, at the University of Adelaide, Waite Campus. Participants rated their liking and intensity of their emotional responses for each sample. The unoaked Chardonnay warm-up sample was presented first for both sessions. The statistical analyses did not show any significant differences between the two sessions in the evaluation of the warm-up sample indicating that wines were evaluated in a similar manner in both sessions.

Four participants failed to complete the Fine Wine Instrument questions; therefore, their data were excluded from analysis, resulting in a final number of participants of 122.

### 2.3. Study 2 (Shiraz Wines)

#### 2.3.1. Wine Samples

One hundred commercially available Australian Shiraz wines were assessed by 8 expert judges (wine expert selection was based on the criteria outlined by Parr et al. [48]) and placed consensually into one out of 4 quality categories—gold (an exceptional wine), silver (an extremely good wine), bronze (a good wine) medal and no medal but of sound quality (no faults present)—following the Australian wine show system [50]. Based on the wine experts’ recommendations, sensory properties and availability, three wines from each of the four quality levels were selected for the consumer tasting.

#### 2.3.2. Consumer Tasting, 3 Trials, 3 Different Contexts (Sensory Lab, Home and Restaurant)

A total of 349 regular Shiraz red wine consumers (who consumed Shiraz wine at least once a month) participated in one of three consumer trials. Each trial had a different set of four wines. Within each trial, the consumers tasted the same four wines, one of each quality level, under three contexts (sensory laboratory, restaurant, home). In the sensory laboratory, consumers evaluated wines in individual booths. Then, 30 mL of wine samples were presented in coded standard ISO glasses sequentially and monadically in randomised order. Water and unsalted crackers were provided for palate cleansing between samples. In the restaurant, participants were given 30 mL wine samples in coded ISO glasses in randomised order and instructed to evaluate all samples over the course of the dinner in that order. Consumers could bring a friend or family member to accompany them. For the home use test, the four wines in 750 mL wine bottles were given to the participants either after the previous tasting session or posted together with the home use questionnaire including detailed instructions. The wines for the home use tasting were masked in such a way that no identifying information about the wines was available to the respondents. Participants were instructed to taste all four samples on the same day with a main meal and to taste the wines in such a way as they would usually consume wine at home (e.g., use own glasses, preferred serving temperature, amount poured). The order of the tasting context was randomised across participants and they were not aware that they were tasting the same wines in the different contexts.

Additionally, to investigate differences in FWS mood in anticipation of the wine tastings, participants’ moods were captured using the Brief Mood Introspection Scale (BMIS) [51] before tasting and evaluated liking, emotional responses and willingness to pay for the tasted wines. Three participants failed to complete the Fine Wine Instrument questions; therefore, their data were excluded from analysis, resulting in a final number of 346 participants.

### 2.4. Data Analyses

Agglomerative Hierarchical Clustering (AHC) using Squared Euclidean distance and Ward’s method was used to categorise consumers into one of the three identified fine wine segments (FWS) (No Frills, Aspirants and Wine Enthusiasts) based on their responses to the Fine Wine Instrument scale [8]. Demographic data were analysed by Chi-square and z-test to investigate significant differences between FWS.

Repeated Measures Analysis of Variance (rANOVA), with wine sample and information condition as within-subject effects and FWS as between-subject factor, was used to analyse consumers’ liking and emotional responses in Study 1. Previously, Danner et al. [47] showed that the inclusion of a trial as a random effect does not improve the ANOVA model, so a similar rANOVA approach with wine quality and context as within-subject effects and FWS as between-subject factor was chosen to analyse liking and emotion ratings of Study 2.

All statistical analyses were performed with SPSS Statistics (Version 25, IBM Corporation, New York, NY, USA) at 5% level of significance.

## 3. Results and Discussion

The results and discussion section focusses on the differences between consumer segments. For detailed results of the total cohort see Danner et al. [47] for Study 1 and Danner et al. [41] for Study 2, respectively.

### 3.1. Fine Wine Segments

In both studies, the AHC classified the participants into three groups, based on their responses to the FWI scale (Table 1). Wine Enthusiasts scored highly on all three dimensions (Connoisseur, Knowledge and Provenance), No Frills comparably lower and the Aspirants ranged in between, scoring close to the midpoint of the scale. The values of the cluster centroids (geometric centres), especially of the No Fills, were slightly higher in both studies compared to the original Johnson and Bastian [8] study. This may be because of the different data collection methods used. Johnson and Bastian [8] used an online survey for data collection, whereas the present studies collected data at the university sensory lab or restaurant, which required a greater effort on the part of the respondents to participate and therefore may have biased the sample towards more wine-involved and knowledgeable participants compared to the online studies and general public. In future studies of this kind, recruitment could take place in restaurants, cafes, or hotels where patrons are consuming wine. Less involved wine consumers may then be included in the respondents.

### 3.2. Demographic Composition of the Fine Wine Segments

Table 2 summarises the demographic composition of the three different FWS segments for both studies. The results of the Chi-square test showed no significant differences (*p* < 0.05) between FWS for participants’ gender, education, or household income for both studies. In Study 2, significant differences were found for age, indicating that a higher proportion of Wine Enthusiasts were older than 65 compared to the No Frills segments. This result differed from that found by Johnson and Bastian [8], whose Wine Enthusiast segment had almost 40% under the age of 35. Once again, this disparity may be due to the data collection methods employed in the two studies.

The results of the Chi-square test for wine consumption frequency are not reported for Study 1 as the expected cell count was lower than 5 for 6 cells and, therefore, the results are not reliable; however the z-test indicated that Wine Enthusiasts consumed wine more frequently compared to No Frills. Similar results were found in Study 2 and in agreement with previous studies [8,14,16,26]. Moreover, in agreement with earlier literature, it was found that the more involved segments spent significantly more money on wine compared to the No Frills segment [16,52].

### 3.3. Research Question 1: Fine Wine Segments’ Hedonic Responses When Tasting Wine in Different Contexts

#### 3.3.1. Study 1 (White Wines)

Results of the rANOVA show significant main effects of sample (F(1.633, 194.313) = 21.258, *p* < 0.001), information context (F(1.706, 204.041) = 37.068, *p* < 0.001) and FWS (F(2, 119) = 4.305, *p* = 0.016) on liking as well as a significant interaction effect of sample*FWS (F(4, 238) = 2.797, *p* = 0.027) (Table 3). Post-hoc comparisons showed that Sauvignon Blanc and Riesling were liked significantly more than Chardonnay across all three information conditions and all wines were liked most in the elaborate information condition and least in the blind tasting with the basic description in between. These results accord with the findings of King et al. [53] who reported that their Australian respondents significantly preferred Sauvignon Blanc and Riesling varieties over oaked Chardonnay.

Regarding the FWS, the results showed that Wine Enthusiasts liked the wines significantly more ($\bar{x}$ = 6.7) compared to the No Frills segment ($\bar{x}$ = 6.0), with no significant difference observed for Aspirants ($\bar{x}$. = 6.5). These findings are in agreement with Verdonk et al. [27], who found that their identified Wine Enthusiasts liked five out of six sparkling wines styles significantly more compared to their other two segments.

**Figure 1:**
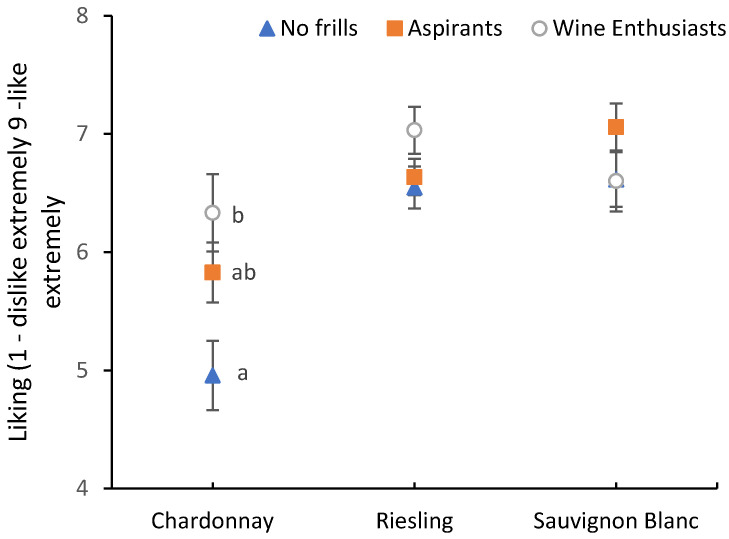
Hedonic responses of the fine wine segments (FWS) towards the three wine samples, pooled for information conditions. Liking ratings were measured on a 9-point hedonic-scale, ranging from 1 = dislike extremely to 9 = like extremely. Error bars indicate standard errors. Different lowercase letters indicate significant differences between FWS based on Fishers least significant difference (LSD) post-hoc comparisons.

Investigating the significant sample*FWS interaction (Figure 1), the higher liking ratings of the Wine Enthusiasts were predominantly driven by an increased liking of the Chardonnay sample, with no significant differences between FWS for Riesling and Sauvignon Blanc. A possible explanation for this effect might be that less involved consumer segments are less familiar with the sensory characteristics of the oaked and aged Chardonnay sample. King et al. [53] identified a segment of consumers in their study that they labelled “Chardonnay wine dislikers”. This segment was female predominant, with low levels of wine knowledge and wine involvement and consumed more Sauvignon Blanc than other white wine varieties. Our No Frills and Aspirant segments also had more female than male members. It may be that these female respondents were similarly less familiar with Chardonnay wines, supporting the results of King et al. [53]. An examination of the types/styles of wines consumed by the three FWS showed that there were no significant differences between the segments around their consumption of Riesling, Sauvignon Blanc and Chardonnay. However, there was a trend for Wine Enthusiasts to consume more Chardonnay and less of the other two varieties, compared to the No Frills and Aspirants segments (data not shown). This lends some weight to the argument that the Wine Enthusiasts segment was more familiar with Chardonnay wine as a category, than the other two segments and is consistent with the findings of Johnson and Bastian [8], whose Wine Enthusiasts’ segment consumed significantly more oaked Chardonnay than the other two segments.

Although it has previously been suggested that highly involved wine tasters prefer more elaborate wine descriptions compared to less experienced or involved tasters [54], no significant information*FWS interaction was observed, indicating that the three different FWS’s hedonic responses were influenced by the three information conditions in a similar way. These findings are in agreement with a more recent study by Mueller et al. [55] who found no significant relationship between consumers’ level of involvement and the relative influence of wine back label information on their wine choice.

#### 3.3.2. Study 2 (Shiraz Wines)

**Figure 2:**
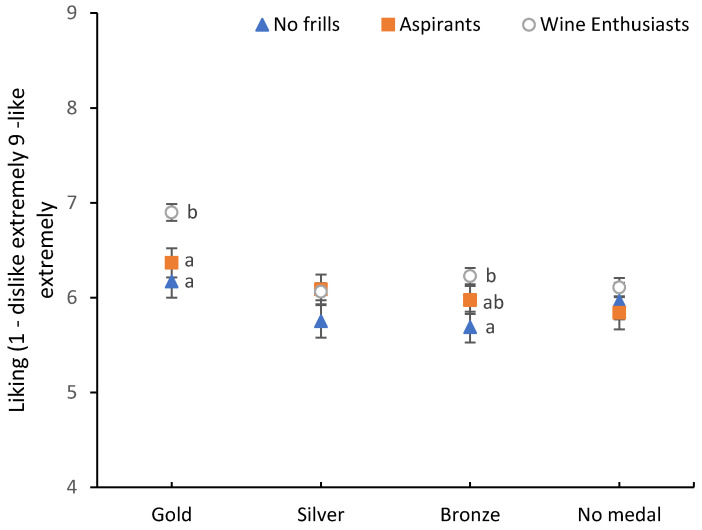
Hedonic responses of the fine wine segments (FWS) towards the four wine quality levels, pooled for context. Liking ratings were measured on a 9-point hedonic-scale, ranging 1 = dislike extremely to 9 = like extremely. Error bars indicate standard errors. Different lowercase letters indicate significant differences between FWS based on Fishers LSD post-hoc comparisons.

rANOVA showed significant main effects of quality (F(2.748, 942.626) = 12.301, *p* < 0.001) and FWS (F(2, 343) = 3.813, *p* = 0.023), but not for consumption context (F(2, 686) = 0.288, *p* = 0.750) on liking (Table 4). Post-hoc comparisons for the main effects, showed that gold medal wines were liked significantly more compared to the three lower quality levels, with no significant differences between the latter three. Wine Enthusiasts liked the wines more ($\bar{x}$ = 6.3) compared to No Frills ($\bar{x}$ = 5.9), with no significant differences to Aspirants ($\bar{x}$ = 6.1). Figure 2 indicates that this effect is predominantly driven by the higher liking ratings of gold medal wines by the Enthusiasts, and to a lesser extent by bronze medal wines. It could be that the higher amounts spent when purchasing wine by the Wine Enthusiasts exposed them to higher quality or more complex wines and that exposure might explain their higher hedonic response to the Gold medal wines. This could indicate that the Wine Enthusiasts are displaying similar preferences to the wine experts, consistent with previous findings by Johnson [56], who found that liking ratings of highly-involved consumers may reflect wine experts’ opinions.

Similar to Study 1 no significant context*FWS interaction was observed, indicating that the three different FWS’s hedonic responses were influenced by the consumption conditions in a similar way.

### 3.4. Research Question 2: Fine Wine Segments’ Emotional Responses When Tasting Wine in Different Contexts

#### 3.4.1. Study 1 (White Wines)

The results of the rANOVAs are summarized in Table 3, showing significant sample effects on 15 out of 19 emotions. Independent from information conditions, significantly more intense positive emotions and less intense negative emotions were reported for Sauvignon Blanc compared to the Chardonnay sample, with the Riesling sample mostly ranging in between. Significant information effects were observed on 14 emotions. More intense positive and less intense negative emotions were reported for wines tasted under the elaborate information condition compared to the blind condition, the basic (sensory information only) condition ranged in-between. For more details on the unsegmented results, see Danner et al. [41].

**Figure 3:**
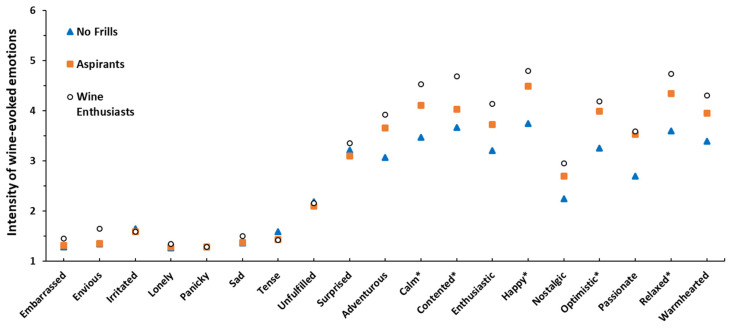
Reported wine evoked emotions for the 3 fine wine segments (FWS), pooled for information condition and samples. Emotion intensities were measured on a 9-point scale, ranging from 1 = not at all to 9 = extremely. * indicates significant differences between FWS at *p* < 0.05.

Examination of the reported evoked emotions between FWS, revealed significant differences for five positive emotions *calm*, *happy*, *optimistic*, *relaxed* and *warm-hearted*, but no significant interactions between FWS and sample or FWS and information condition. Figure 3 shows that independent of wine variety and information condition, more intense positive emotions were reported by the Wine Enthusiasts followed by Aspirants and No Frills. No significant effect on any negative emotions was observed. This aligns with the results of Calvo-Porral et al. [46], who segmented consumers based on their emotional associations with wine. Their “wine lovers”, the segment with the most intense positive emotional associations, were also significantly more wine-involved compared to the “unattached” who stated significantly less intense positive emotional associations. These segments also shared similar wine consumption frequency trends with their FWS counterparts; “wine lovers” and Wine Enthusiasts were consuming wine more frequently compared to the “unattached” and No Frills segments, respectively.

#### 3.4.2. Study 2 (Shiraz)

Table 4 summarises the results of the rANOVAs. Out of 19 emotions, significant effects of wine quality were observed for 10. Independent of consumption context, more intense positive emotions were reported for gold medal wines compared to samples of lower quality. Similar effects of decreasing intensity of positive emotions with reduced wine quality, were found by Jiang et al. [45] who altered green aromas, and Niimi et al. [57] who increased astringency in wine; both attributes often associated with wine quality. In addition, both studies also saw an increase in negative emotions not found in the present study. Context had a significant effect on 11 out of the 19 emotions, with participants feeling more positive emotions (e.g., *calm*, *contented*, *enthusiastic*, *happy* and *relaxed*) in the restaurant compared to the lab, with the at home context ranging in between.

**Figure 4:**
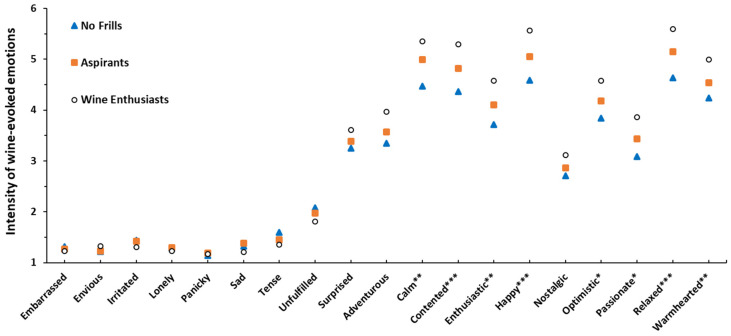
Reported wine evoked emotions for the 3 fine wine segments (FWS), pooled for context and samples. Emotion intensities were measured on a 9-point scale, ranging from 1 = not at all to 9 = extremely. * indicates significant differences between FWS at *p* < 0.05, ** at *p* < 0.01 and *** at *p* < 0.001.

**Figure 5:**
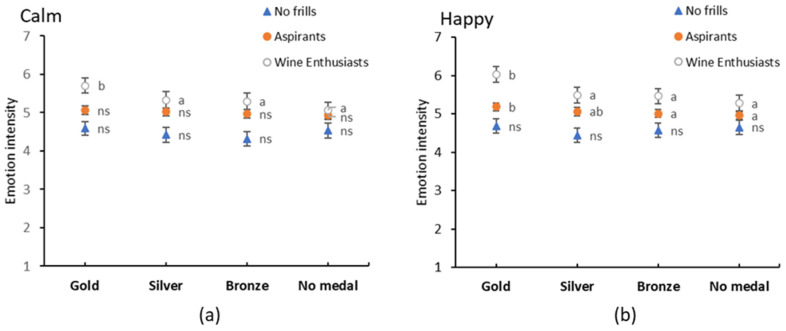
Interaction between fine wine segments (FWS) and quality for (**a**) calm and (**b**) happy emotions. Error bars indicate standard errors. Different lowercase letters indicate significant differences between wine quality within a FWS based on Fishers LSD post-hoc comparisons, ns—no significant differences.

Emotional responses significantly differed for eight positive emotions between FWS, with the Wine Enthusiasts revealing more intense positive emotions of *calm*, *contented*, *enthusiastic*, *happy*, *optimistic*, *passionate*, *relaxed* and *warm hearted* compared to the No Frills segment, with Aspirants ranging in between, confirming the results of Study 1 and Calvo-Porral et al. [46] (Figure 4). Similar to Study 1, no significant differences were observed in negative emotions. Investigation of the interactions between FWS and quality uncovered significant effects for three emotions, *calm*, *happy* and *embarrassed*. Closer examination of the post-hoc comparisons (Figure 5) show that Wine Enthusiasts’ emotions of *happy* and *calm* were particularly high for Gold medal wines, whereas Aspirants’ and No Frills’ emotions were consistent across the quality levels, which aligns with their hedonic ratings. Despite being significant, the effect on *embarrassed* was considered unimportant as the intensity ratings only ranged between 1.13 and 1.4 on a 9-point scale.

**Figure 6:**
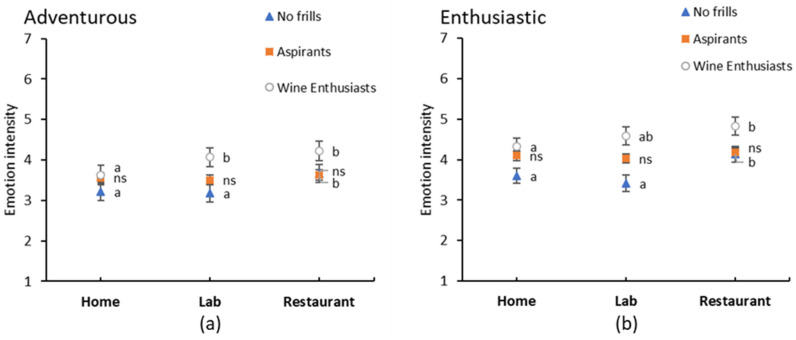
Interaction between fine wine segments (FWS) and context for (**a**) adventurous and (**b**) enthusiastic emotions. Error bars indicate standard errors. Different lowercase letters indicate significant differences between context within a FWS based on Fishers LSD post-hoc comparisons, ns—no significant differences.

Interestingly, significant interaction effects between FWS and context were observed for two emotions, *adventurous* and *enthusiastic* which is in contrast to the liking and emotion results of Study 1. Closer examination showed that Aspirants rated both emotions similarly in all three consumption contexts, whereas Wine Enthusiasts and No Frills state more intense emotions of *adventurous* and *enthusiastic* in the restaurant compared to at home (Figure 6). Furthermore, Wine Enthusiasts tended to be more *adventurous* and *enthusiastic* in the laboratory compared to the No Frills, indicating that they generally enjoy wine more than the No Frills segment, regardless of the consumption context. To gain a deeper understanding of why the segments react differently to context, future research could investigate wine-related behaviours such as restaurant consumption, or even personality traits, similar to Mora et al. [58].

### 3.5. Fine Wine Segments’ Mood in Anticipation of Tasting Wines in Different Contexts

**Figure 7:**
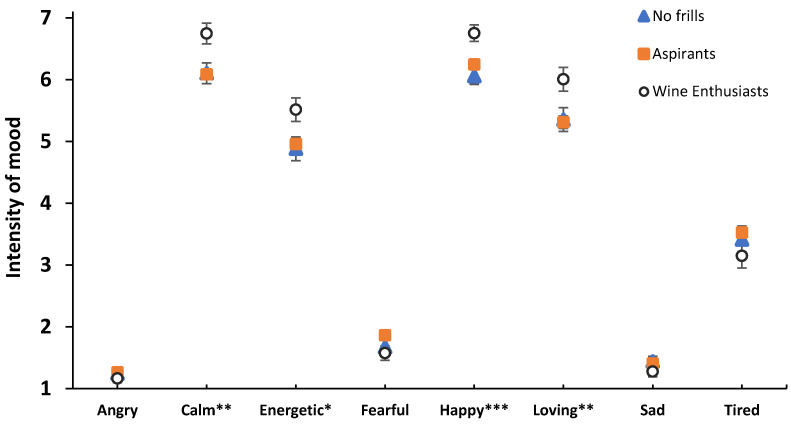
Consumers’ moods by fine wine segments (FWS) prior to tasting the samples. Results pooled for context. Mood intensities were measured on a 9-point scale, ranging from 1 = not at all to 9 = extremely. Error bars indicate standard error. * indicates significant differences between FWS at *p* < 0.05, ** at *p* < 0.01 and *** at *p* < 0.001.

To investigate differences in FWS mood in anticipation of the wine tastings, before commencing any wine tasting, consumers were asked to rate their moods using the Brief Mood Introspection Scale (BMIS) [51] in all three contexts. Results showed no significant interaction between FWS*context for any of the rated moods, suggesting that the different FWS segments reacted in a similar way to the tasting contexts (detailed ANOVA results in Appendix A Table A2). A significant effect of FWS was observed for calm, energetic, happy and loving moods. This indicates that Wine Enthusiasts were not only more emotional when consuming or tasting wines, but that they were also in a more positive mood. This may be because in anticipation of tasting wines, they were more positively engaged with the task, which could reflect that this segment is generally more positive about wine compared to the other segments (Figure 7).

## 4. General Discussion and Conclusions

This is one of the first studies investigating wine consumer lifestyle segmentation while capturing participants’ responses when actually tasting wines, as opposed purely to online self-reported surveys. Across the two studies, it was found that tasting or consuming wine elicits different hedonic (research question 1) and emotional responses (research question 2) in the three identified consumer segments derived from the fine wine instrument (FWI).

The identified Wine Enthusiast segment generally liked the tasted wines more and stated more intense positive emotions when tasting or consuming wine compared to the No Frills segment, with the Aspirants ranging in between. Furthermore, Wine Enthusiasts were more discriminant regarding wines preferred and were more emotional about higher quality wines (Study 1) and more complex wines (aged and oaked Chardonnay, Study 2). Therefore, if wine quality and complexity are of interest to wine producers, it might be beneficial to focus research efforts on Wine Enthusiasts. In summary, these results indicate that “domain-specific” lifestyle segmentation, in this case the FWI, is a reliable segmentation method resulting in meaningful segments.

Interestingly, although significant main effects of context were observed for both studies, these effects were consistent for all three consumer segments, with only minor differences in the emotional responses of *adventurous* and *enthusiastic*, indicating that FWS are similarly influenced by the tested contexts and information conditions. A possible explanation could be that consumers were only segmented on their responses to the fine wine instrument. If another segmentation base encompassing other lifestyle components was chosen, the impact of context and information conditions might differ.

Consistently across both studies, negative emotions were only elicited to a very small extent, and no significant differences were observed between FWS. This is in agreement with previous studies showing that commercial products of sound quality are generally associated with moderately intense positive emotions, but related very little with negative emotions [59,60]. Only if faults or less desirable sensory properties are exaggerated, do negative emotions increase in intensity and discriminate between samples, e.g., intense astringency [57] or green aroma [45]. Although the quality of Shiraz wines in Study 2 varied, even the “No medal” wines were of sound quality and no faults or off-flavours were present.

It must be noted that data were collected exclusively in Australia and, although segmentation outcomes show considerable overlap with other markets (e.g., Spain [46] or South Africa [26]), further studies are required to confirm that the results are transferable to other countries and markets.

Recently, Mora et al. [58] segmented consumers based on their personality using the Big Five Inventory questionnaire (BFI) [61] and found differences in hedonic and emotional responses between the personality segments when tasting a wide range of different wines. Investigating the personality traits of the identified FWS could be of high interest for future research and might assist in gaining a better understanding of how consumers’ wine knowledge and involvement influence purchase decisions.

Wine involvement has often been associated with wine knowledge. The current studies, in agreement with Calvo-Porral et al. [46], also show a significant emotional aspect to wine consumers’ involvement. Therefore, creating positive emotional experiences around wine might be a promising opportunity to increase consumers’ wine involvement.

## Figures and Tables

**Table 1:** Cluster centroids for the three fine wine segments following the Agglomerative Hierarchical Clustering (AHC).

	Fine Wine Consumer Segment					
	Study 1 (*n* = 122)			Study 2 (*n* = 346)		
	No Frills	Aspirants	Wine Enthusiasts	No Frills	Aspirants	Wine Enthusiasts
Dimension	*n* = 39; 32%	*n* = 52; 43%	*n* = 31; 25%	*n* = 72; 21%	*n* = 215; 62%	*n* = 59; 17%
Connoisseur	4.5 ^a^	5.8 ^b^	7.0 ^c^	4.2 ^a^	5.7 ^b^	7.2 ^c^
Knowledge	4.0 ^a^	5.9 ^b^	7.3 ^c^	3.8 ^a^	5.3 ^b^	7.2 ^c^
Provenance	4.5 ^a^	5.8 ^b^	6.5 ^c^	3.9 ^a^	5.6 ^b^	6.9 ^c^

^a^, ^b^ and ^c^ indicate significant differences between segments at *p* < 0.05 based on Tukey corrected post-hoc comparisons.

**Table 2:** Demographic data and wine consumption frequency of the three fine wine segments.

	Fine Wine Consumer Segment							
	Study 1				Study 2			
	No Frills	Aspirants	Wine Enthusiasts	Total	No Frills	Aspirants	Wine Enthusiasts	Total
	*n* = 39; 32%	*n* = 52; 43%	*n* = 31; 25%	*n* = 122	*n* = 72; 21%	*n* = 215; 62%	*n* = 59; 17%	*n* = 346
	[%]	[%]	[%]	[%]	[%]	[%]	[%]	[%]
*Gender*								
Male	46.3	41.5	53.1	46.0	54.2	55.3	62.7	56.4
Female	53.7	58.5	46.9	54.0	45.8	44.7	37.3	43.6
*Age*								
18–34	17.1	26.4	15.6	20.6	25.0	20.0	13.6	19.9
35–49	31.7	18.9	15.6	22.2	29.2	29.8	22.0	28.3
50–64	29.3	34.0	46.9	35.7	37.5	34.0	40.7	35.8
65+	22.0	20.8	21.9	21.4	8.3 ^a^	16.3 ^ab^	23.7 ^b^	15.9
*Education*								
No tertiary	48.8	34.0	53.1	43.6	50.0	40.9	49.2	44.2
Bachelor degree	19.5	30.2	21.9	24.6	29.2	30.7	23.7	29.2
Postgraduate degree	31.7	35.8	25.0	31.8	20.8	28.4	27.1	26.6
*Household Income [AUD]*								
<50,000	14.6	18.9	21.9	18.3	19.4	13.0	20.3	15.6
50,001–100,000	41.5	45.3	37.5	42.0	40.3	40.5	35.6	39.6
100,001–200,000	34.1	26.4	31.3	30.2	38.9	39.5	35.6	38.7
>200,000	9.8	9.4	9.4	9.5	1.4	7.0	8.5	6.1
*General Wine Consumption Frequency*								
Few times per week	65.9 ^a^	69.8 ^ab^	87.5 ^b^	73.0	61.1 ^a^	75.8 ^b^	78.0 ^b^	73.1
Once a week	12.2	17.0	9.4	13.5	22.2	16.3	16.9	17.6
Once every 2 weeks	14.6	11.3	3.1	10.3	13.9 ^b^	5.1 ^a^	3.4 ^a^	6.6
Once a month	7.3	1.9	0.0	3.2	2.8	2.8	1.7	2.6
*Average Spent for a Bottle of Wine [AUD]*								
For home consumption	15.3 ^a^	18.8 ^b^	17.4 ^a b^		17.1 ^a^	18.8 ^a^	23.2 ^b^	
A a restaurant	35.7 ^a^†	39.1 ^a b^†	42.3 ^b^†		33.8 ^a^	37.8 ^b^	44.1 ^c^	
For a special occasion	34.0 ^a^	38.3 ^a b^	41.5 ^b^		NA ‡	NA ‡	NA ‡	

Different lower-case letters indicate significant differences at *p* < 0.05 based on z-test for frequency and Tukey corrected post-hoc comparisons for continuous data; † *n* = 122 as 4 participants stated that they do not regularly purchase bottles of wine in restaurants.; ‡ not asked in Study 2. Australian Dollar (AUD).

**Table 3:** Results of the Repeated Measures ANOVA investigating effects of sample (S), information (I) and fine wine segments (FWS) on liking and reported product-evoked emotions for Study 1.

	S	I	FWS	S*FWS	I*FWS	S*I	S*I*FWS
	*p*	*p*	*p*	*p*	*p*	*p*	*p*
Liking	**<0.001**	**<0.001**	**0.016**	**0.027**	0.598	0.715	0.430
Adventurous	**<0.001**	**0.001**	0.096	0.296	0.284	0.917	0.651
Calm	**0.002**	**0.007**	**0.019**	0.428	0.321	0.597	0.590
Contented	**<0.001**	**0.009**	**0.022**	0.112	0.796	0.255	0.170
Embarrassed	**0.004**	0.060	0.464	0.738	0.607	0.891	0.180
Enthusiastic	**<0.001**	**0.001**	0.066	0.059	0.185	**0.042**	0.742
Envious	0.633	0.662	0.208	0.609	**0.002**	0.110	0.772
Happy	**<0.001**	**<0.001**	**0.017**	0.320	0.091	0.555	0.788
Irritated	**<0.001**	**0.011**	0.927	0.149	0.220	0.601	0.115
Lonely	0.402	0.569	0.871	0.767	0.645	**0.047**	0.714
Nostalgic	0.886	**<0.001**	0.150	0.100	0.094	0.480	0.777
Optimistic	**0.008**	**<0.001**	**0.045**	0.411	0.088	0.629	0.660
Panicky	**0.008**	0.454	n.a.	0.296	0.269	0.872	0.074
Passionate	**0.003**	**<0.001**	0.052	0.147	0.401	0.700	0.186
Relaxed	**0.001**	**0.004**	**0.012**	0.103	0.210	0.495	0.216
Sad	**<0.001**	0.458	0.645	0.835	0.935	0.647	0.795
Surprised	0.129	**0.024**	0.791	0.693	0.387	0.082	0.202
Tense	**<0.001**	**0.009**	0.520	0.245	0.650	0.560	0.678
Unfulfilled	**<0.001**	**<0.001**	0.935	0.106	0.955	0.687	0.825
Warm-hearted	**0.004**	**<0.001**	0.067	0.100	0.339	0.891	0.684

Bolded values indicate significant effects at *p* < 0.05.

**Table 4:** Results of the Repeated Measures ANOVA investigating effects of quality (Q), context (C) and fine wine segments (FWS) on liking and reported product-evoked emotions for Study 2.

	Q	C	FWS	Q*FWS	C*FWS	C*Q	Q*C*FWS
	*p*	*p*	*p*	*p*	*p*	*p*	*p*
Liking	**<0.001**	0.750	**0.023**	0.114	0.749	0.219	0.548
Adventurous	0.305	**<0.001**	0.095	0.721	**0.019**	0.059	0.156
Calm	**<0.001**	**<0.001**	**0.002**	**0.032**	0.576	0.476	0.491
Contented	**<0.001**	**0.005**	**<0.001**	0.194	0.481	0.069	0.822
Embarrassed	0.553	**0.047**	0.585	**0.001**	0.850	**0.031**	0.767
Enthusiastic	**0.001**	**<0.001**	**0.004**	0.240	**0.008**	0.354	0.999
Envious	0.891	0.685	0.473	0.538	0.449	0.255	0.124
Happy	**<0.001**	**<0.001**	**<0.001**	**0.022**	0.075	0.227	0.831
Irritated	0.172	0.276	0.376	0.704	0.428	0.227	0.512
Lonely	0.763	0.347	0.684	0.135	0.262	0.242	0.512
Nostalgic	**0.001**	0.348	0.300	0.067	0.501	0.813	0.737
Optimistic	**0.016**	**0.002**	**0.024**	0.209	0.260	0.125	0.305
Panicky	0.612	0.736	0.716	0.885	0.953	0.887	0.912
Passionate	**<0.001**	**0.011**	**0.029**	0.524	0.667	0.872	0.995
Relaxed	**0.001**	**<0.001**	**<0.001**	0.300	0.364	0.547	0.649
Sad	0.576	0.561	0.131	0.823	0.661	0.867	0.806
Surprised	**<0.001**	0.534	0.382	0.148	0.330	0.144	0.561
Tense	0.246	**0.002**	0.077	0.907	0.669	0.909	0.431
Unfulfilled	0.103	0.302	0.265	0.138	0.652	0.708	0.351
Warm-hearted	**<0.001**	**<0.001**	**0.009**	0.101	0.516	0.793	0.658

Bolded values indicate significant effects at *p* < 0.05.

## Appendix A

**Table A1:** The three dimensions of the Fine Wine Instrument (FWI) with their respective scale items [8].

**Connoisseur Dimension**
I can generally recall the memorable wines that I drink.
I keep a record of the wines that I buy.
I prefer shopping for and buying wine from specialty outlets.
I have a special wine storage space (either at home or elsewhere) that allows me to age my wines and maintain a wine collection.
I usually buy at least a half dozen bottles (mixed or same) each time I buy wine.
I always check my wine for cork or other taints.
I prefer to drink older wines than younger wines.
**Knowledge Dimension**
I regularly attend special wine tastings or wine club meetings.
I regularly read wine magazines and wine reviews in newspapers.
I take more notice of wine related articles in the press and TV than I did two years ago.
Being knowledgeable about wine gives me a great deal of satisfaction.
I would like to learn more about wine styles and their countries of origin.
**Provenance Dimension**
I am choosy when it comes to selecting wines from particular vintages.
I often look for rare or scarce wines.
For me, the grape variety from which the wine is made is an important consideration.
I prefer wines from certain geographical regions.
In my wine collection, it is important to have wines from countries other than Australia.
When drinking wine, it is important for me to know in which country the wine was made.

**Table A2:** Results of the Repeated Measures ANOVA investigating effects of context (C) and Fine Wine Segment (FWS) on consumers moods in Study 2.

Moods	C	FWS	C*FWS
	*p*	*p*	*p*
Angry	0.659	0.398	0.656
Calm	0.231	**0.002**	0.486
Energetic	0.072	**0.022**	0.981
Fearful	**0.003**	0.052	0.848
Happy	**<0.001**	**<0.001**	0.913
Loving	**0.001**	**0.006**	0.524
Sad	0.879	0.309	0.530
Tired	0.843	0.231	0.975

Bolded values indicate significant effects at *p* < 0.05.
